# Real-time gastric polyp detection using convolutional neural networks

**DOI:** 10.1371/journal.pone.0214133

**Published:** 2019-03-25

**Authors:** Xu Zhang, Fei Chen, Tao Yu, Jiye An, Zhengxing Huang, Jiquan Liu, Weiling Hu, Liangjing Wang, Huilong Duan, Jianmin Si

**Affiliations:** 1 Key Laboratory for Biomedical Engineering of Ministry of Education, College of Biomedical Engineering & Instrument Science, Zhejiang University, Hangzhou, China; 2 Institute of Gastroenterology, Zhejiang University, Hangzhou, China; 3 Department of Gastroenterology, Second Affiliated Hospital of Zhejiang University School of Medicine, Hangzhou, China; 4 Department of Gastroenterology, Sir Run Run Shaw Hospital, Medical School, Zhejiang University, Hangzhou, China; Alma Mater Studiorum University of Bologna, ITALY

## Abstract

Computer-aided polyp detection in gastric gastroscopy has been the subject of research over the past few decades. However, despite significant advances, automatic polyp detection in real time is still an unsolved problem. In this paper, we report on a convolutional neural network (CNN) for polyp detection that is constructed based on Single Shot MultiBox Detector (SSD) architecture and which we call SSD for Gastric Polyps (SSD-GPNet). To take full advantages of feature maps’ information from the feature pyramid and to acquire higher accuracy, we re-use information that is abandoned by Max-Pooling layers. In other words, we reuse the lost data from the pooling layers and concatenate that data as extra feature maps to contribute to classification and detection. Meanwhile, in the feature pyramid, we concatenate feature maps of the lower layers and feature maps that are deconvolved from upper layers to make explicit relationships between layers and to effectively increase the number of channels. The results show that our enhanced SSD for gastric polyp detection can realize real-time polyp detection with 50 frames per second (FPS) and can improve the mean average precision (mAP) from 88.5% to 90.4%, with only a little loss in time-performance. And the further experiment shows that SSD-GPNet has excellent performance in improving polyp detection recalls over 10% (p = 0.00053), especially in small polyp detection. This can help endoscopic physicians more easily find missed polyps and decrease the gastric polyp miss rate. It may be applicable in daily clinical practice to reduce the burden on physicians.

## 1. Introduction

Gastrointestinal (GI) endoscopy is the primary method for detecting and removing polyps [1]. However, despite the advantages of this method, the workload influences the physician’s level of operations. Recent clinical studies have shown that even experienced doctors may miss gastric polyps while conducting a gastroscopy under high workload [2, 3]. Although most gastric polyps are benign and harmless, adenoma polyps may develop into gastric cancers if they are not diagnosed and treated in time. [4]. It is therefore important to study automatic gastric polyp detection, which may help clinicians find lesions and reduce the miss rate [5].

Most previous methods for detecting gastrointestinal polyps used the shape of elliptical features[6–9], texture features [10, 11], color, and position features [12, 13], or combinations of these features [14, 15]. However, these features were usually manually designed. They were not robust and were time-consuming. These methods could not achieve real-time gastric lesion detection and they suffered from a high false positive rate. Moreover, most of these studies focused on detecting polyps in colonoscopies, so it remained an open challenge to realize reliable and accurate gastric polyp detection in real time.

In recent years, GPU usage has greatly increased the speed of calculations. In addition, the proposed ReLU activation function [16], dropout techniques [17], transfer learning [18, 19], and many data augmentation [20] methods have, to some degree, alleviated the lack of labeled data and avoided overfitting of the training models. These factors contribute to the success of convolutional neural networks (CNNs) in many tasks of natural images [21, 22], including improving the accuracy and speed of object detection [23–27]. CNNs have also been introduced into medical image classification and detection tasks [28–31], including endoscopic lesion detection [32, 33]. However, previous studies on polyp detection like those in [34, 35] were actually polyp classification instead of detection. For detection, one wants information on the polyp’s location. Among various object detection methods, single shot multibox detector (SSD) [23] is relatively fast and robust under scale variations because it makes full use of multiple convolution layers for object detection and it performs well in both speed and detection accuracy, which is promising for real-time polyp detection. SSD architecture has been used to train endoscopic images for gastric cancer detection [36], which, however, did not alter any detail of SSD. The results of MICCAI 2015 Endoscopic Vision Challenge [37] illustrated the comparative evaluation of colonoscopy polyp detection methods. However, none of the methods studied in that paper achieved real-time polyp detection, and they were only evaluated in colonoscopies.

To tackle the problem of automatic gastric polyp detection in real time—the challenge addressed in this paper—we propose an enhanced SSD architecture called SSD for Gastric Polyps (SSD-GPNet). Compared with conventional SSD, there are two enhancements in our network that help improve the mean average precision (mAP) of gastric polyp detection with only a small increase in time cost. First, we reused information that was abandoned by Max-Pooling layers and concatenated that data as extra feature maps for classification and detection in the feature pyramid. Usually, the feature pyramid represents a collection of the layers which are used as the input to the detection layer [38]. Second, in order to fully utilize the relationship between the layers in the feature pyramid without changing the base network that was located close to the input data, we introduced the approach of concatenating feature maps of the lower layers through deconvolution from upper layers.

Our contributions reported in this paper are as follows. First, we propose an enhanced SSD architecture called SSD-GPNet for gastric polyp detection, which, in our experiment, not only improved the mAP of gastric polyp detection, but also improved the mAP of object detection in a natural image dataset, indicating that our methods were more general, and not limited to the use of medical images. Second, we show that small labeled images can also train reliable models without overfitting using data augmentation and transfer learning. Third, we pioneered research on gastric polyp detection and realized automatic gastric polyp detection in real time with high precision, which should help endoscopic physicians find polyps and decrease the rate of misdiagnosis.

## 2. Materials and methods

### 2.1. Data acquisition

To evaluate the performance of our SSD-GPNet, we collected 404 images with gastric polyps (taken from OLYMPUS EVIS LUCERA ELITE CLV-290SL or OLYMPUS EVIS LUCERA ELITE CLV-260SL) from 215 patients who underwent endoscopic examinations at Sir Run Run Shaw Hospital in Zhejiang province in China from January to June 2015. Patients who did not suffering from polyps during this time have been excluded. We were authorized to have access to the gastroscopy images anonymously, which was collected from Sir Run Run Shaw Hospital in Zhejiang province in China. All the patients provided written informed consent for their medical images to be published and used in this research. Our research was approved by the Ethics Committee of Sir Run Run Shaw Hospital, School of Medicine, Zhejiang University. The ethical approval number was 20171018–14. All the collected images contained at least one polyp and were labeled by an experienced endoscopist. To ensure confidentiality, the examination information (e.g., examination date and patient’s name) was removed from the original gastroscopy images. The processed image size was 560×475. We resized these images to 300×300 with the Lanczos-4 interpolation algorithm based on 8×8 area to fit SSD-GPNet input size. We shuffled the images and randomly selected 50 images as the test dataset. Because the number of remaining images was only 354, too small for training, we rotated these 354 labeled images 180 degrees for data augmentation. After effective image augmentation, there were 708 images in total for training and 50 images that had been randomly selected for testing.

### 2.2. Drawbacks of pooling layers

**Fig 1:**
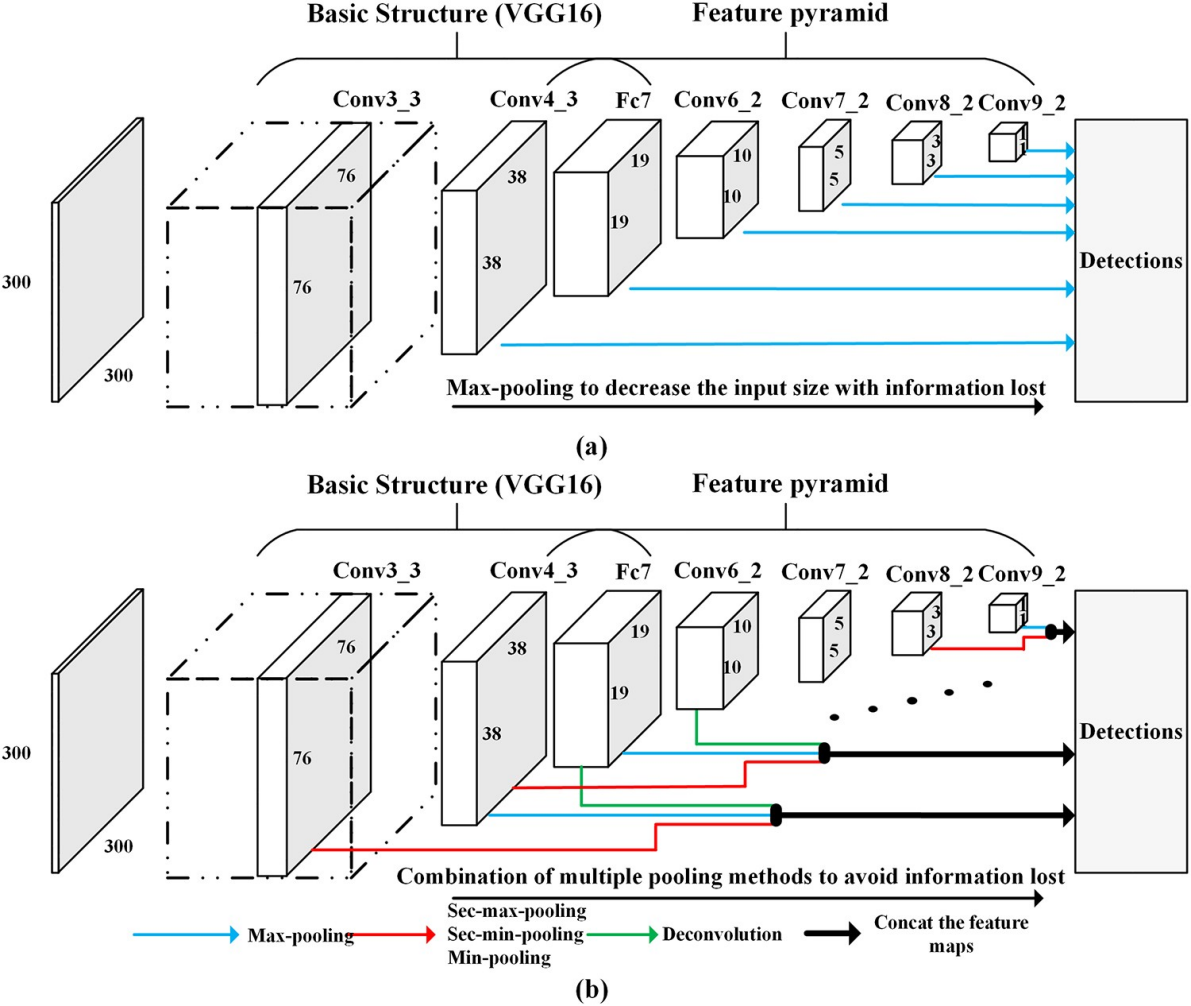
Overall structure of (a) conventional SSD and (b) SSD-GPNet. The layers from Conv4-3 to Conv9-2, which are used as the input to the detections, are denoted as the feature pyramid. Each layer in the feature pyramid is responsible for detecting objects of corresponding size. Blue arrows denote original feature maps for detection. Red arrows represent the use of multiple pooling methods to increase feature maps. Green arrows show the use of deconvolution from neighboring upper layers to increase feature maps. Black arrows represent the concatenation of these feature maps.

**Fig 2:**
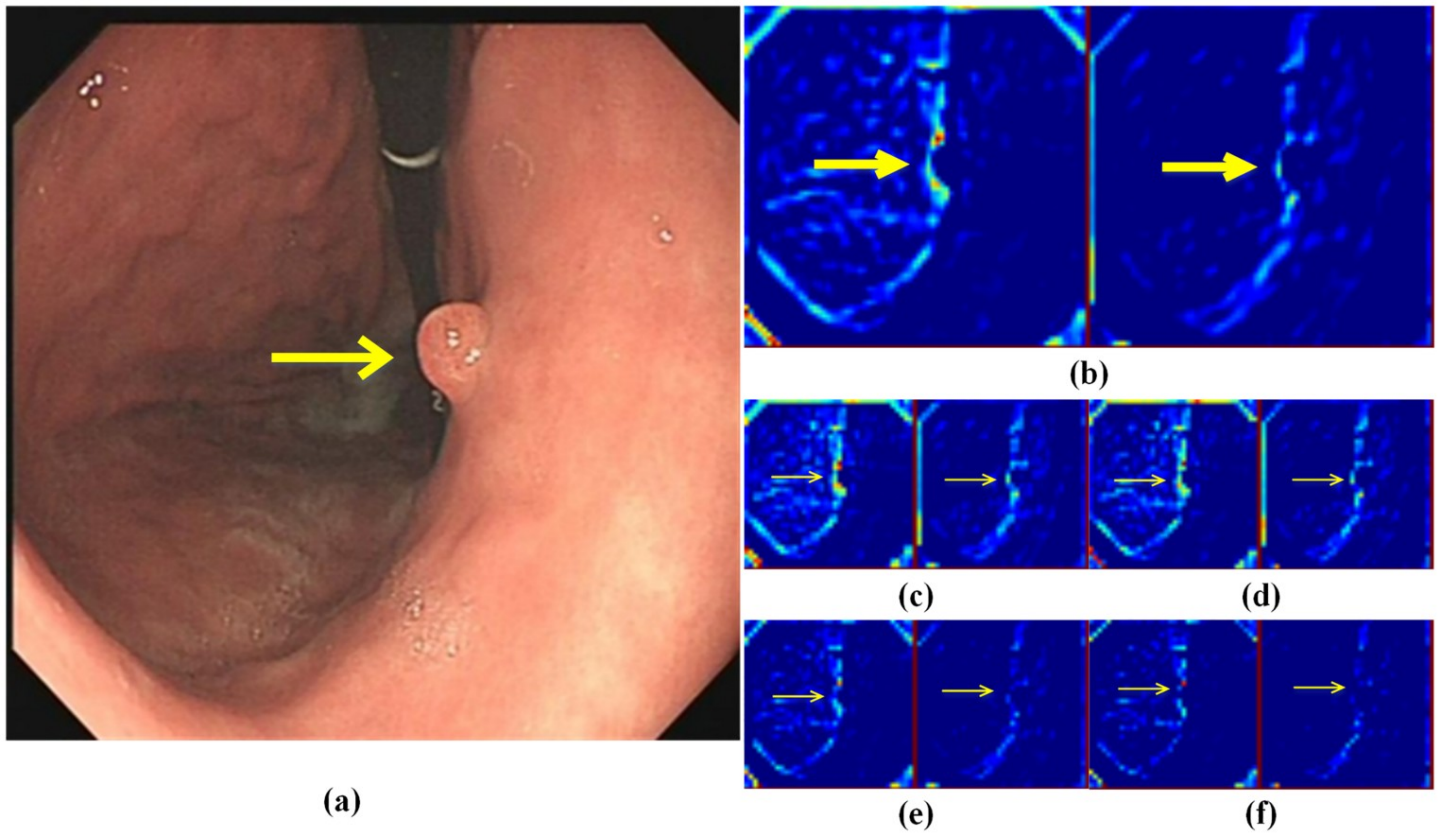
An example to explain that Max-Pooling may cause useful information to be lost. (a) represents an original gastric image with a polyp. (b) denotes two feature maps extracted from Conv3_3 layer. (c), (d), (e), and (f) are maps obtained from (b) using different pooling methods, namely Max-Pooling, Second Max-Pooling, Second Min-Pooling, and Min-Pooling. The yellow arrows denote the polyp location. All the feature maps are pseudo color maps for visualization.

Fig 1(a) shows the conventional SSD architecture. The basic structure is VGG16 [21], following the feature pyramid for object detection. We clearly determined that the feature pyramid reduced the feature map size gradually by pooling layers to reduce the calculations. However, this strategy might lose useful information. For example, from Fig 2, if the kernel size was 2×2 and the stride was 2, after Max-Pooling, we would lose almost 3/4 of the vital information (Fig 2(d), 2(e) and 2(f)), which could also show the features of the polyp shown in the original image (Fig 2(a)).

**Fig 3:**
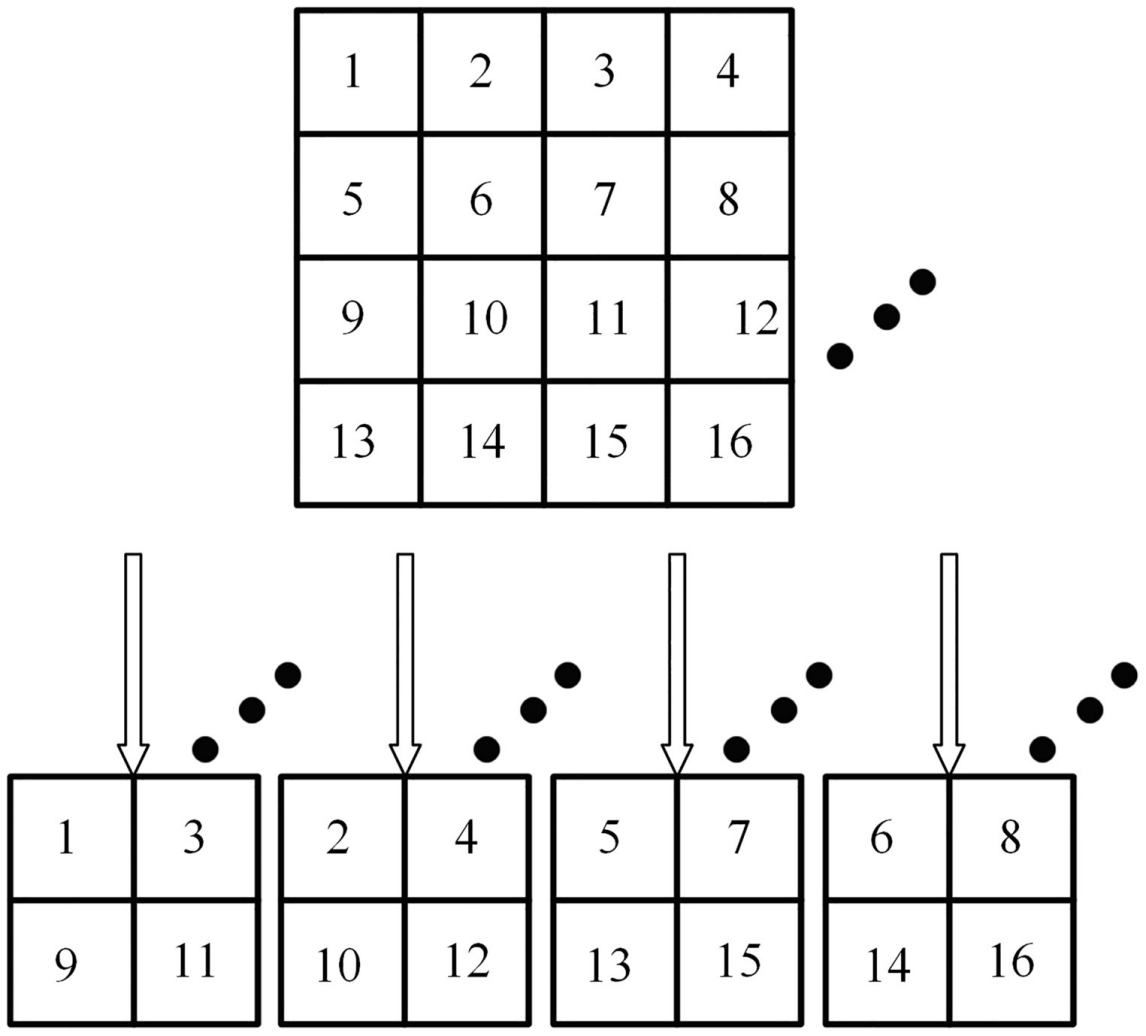
A schematic diagram to illustrate that one feature map can generate four sub feature maps by four different pooling methods. From left to right are indicated the output produced by Min-Pooling, Sec_Min-Pooling, Sec_Max-Pooling, and Max-Pooling respectively.

To avoid this and re-use the information lost from the Max-Pooling layers, we proposed three new pooling layers called Second Max-Pooling (Sec_Max-Pooling), Second Min-Pooling (Sec_Min-Pooling), and Min-Pooling. Fig 3 shows an example that one feature map can generate four sub feature maps by four different pooling methods when the kernel size is 2×2; the padding is zero and the stride is 2.

### 2.3. Pooling module and deconvolution module

To enhance the relationships between layers in feature pyramid, we also introduced the idea mentioned in deconvolutional SSD (DSSD) [39], which applied deconvolution layers to the feature pyramid. Although DSSD increased accuracy, it excessively sacrificed detection speed. To balance speed and accuracy, we simplified the architecture of the feature pyramid proposed in DSSD. We only deconvolved the upper layer for its nearest lower neighbor layer to reduce computation.

**Fig 4:**
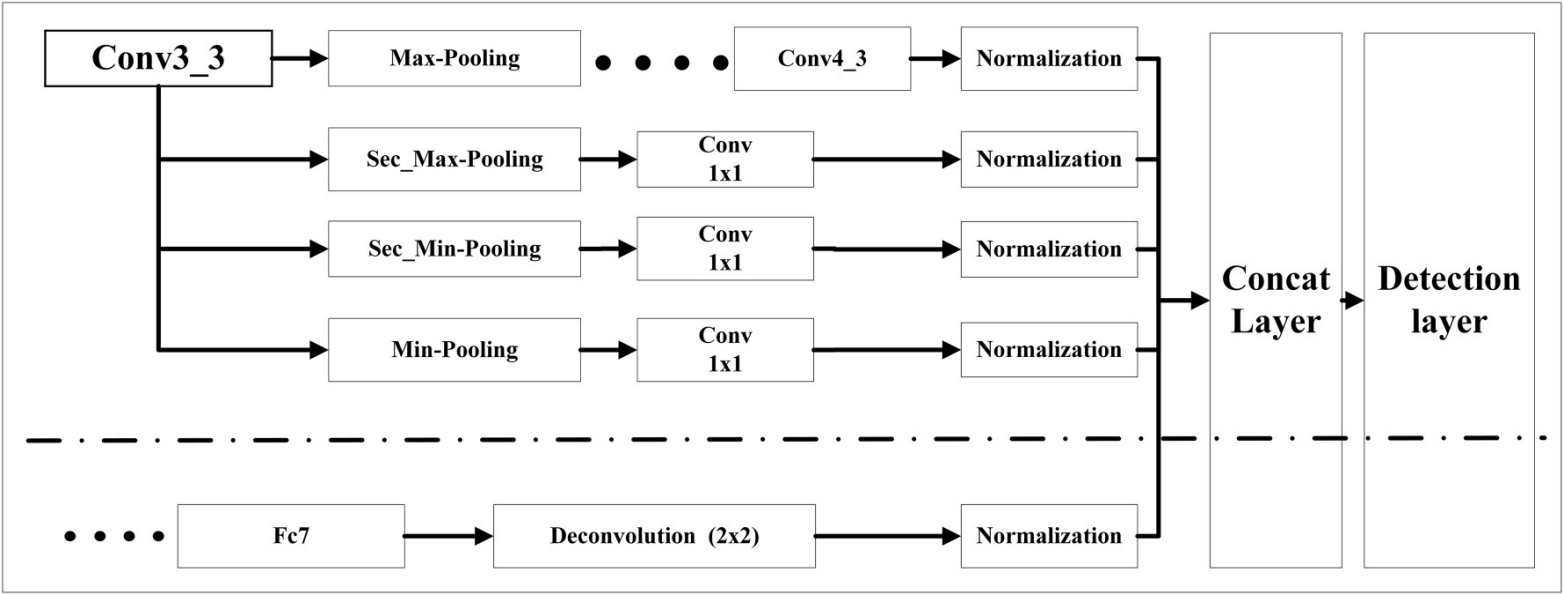
Pooling module and deconvolution module to effectively increase the number of layer Conv4_3’s feature maps. Above the dot-dash line was pooling module. We used multiple pooling methods to increase Conv4_3’s feature maps for detection. Below the dot-dash line was deconvolution module. We adopted deconvolution from Conv4_3’s neighbor feature pyramid layer Fc7 to increase Conv4_3’s feature maps for detection.

Fig 4 shows how we use pooling module and deconvolution module to increase the effective feature maps of Conv4_3, one of feature pyramid’s layers. Above the dot-dash line was pooling module. The added feature maps generated by multiple pooling methods came from the base layer of Conv3_3. Each branch of the pooling layer connected a convolutional layer with 1×1 kernel size. Then a batch normalization layer was adopted after the convolutional layer to ensure that the feature maps were in the same scale before concatenation. Below the dot-dash line was deconvolution module. We up-sampled the layer of Fc7 which was the nearest upper neighbor of Conv4_3 in the feature pyramid with the kernel size of 2×2. Then a batch normalization layer was also adopted. Afterwards, we concatenated the feature maps generated by the pooling and deconvolution modules. The new expanded layer of Conv4_3 was responsible for classification and detection.

### 2.4. The architecture of SSD-GPNet

Similarly, the feature maps of other layers (Fc7, Conv6_2, Conv7_2, Conv8_2, and Conv9_2) in feature pyramid were also expanded like Conv4_3. To achieve the goal of real-time gastric polyp detection, we did not apply the pooling module and deconvolution module to the basic structure to avoid the increase in calculations.

Based on the improvements to the original SSD mentioned above, we proposed an enhanced SSD architecture for gastric polyp detection called SSD-GPNet. The overall structure of SSD-GPNet is shown in Fig 1(b). In particular, because Conv9_2 did not have an upper neighbor layer, it increased its feature maps only by pooling module.

### 2.5. Training

Because of the small set of gastric polyp images, all the experiments reported in this paper used the same pre-trained model, which was downloaded from SSD’s GitHub (https://github.com/weiliu89/caffe/tree/ssd) to initialize model parameters and avoid overfitting. The downloaded model was based on VGG16 and pre-trained on the ILSVRC CLS-LOC dataset like conventional SSD. We applied stochastic gradient descent as the optimizer for the training networks. The training batch size was 8. The basic learning rate was 0.0005, and we have adopted “multi-step” as our learning rate decay policy. The training underwent 100,000 iterations. The learning rate decayed after 50,000 iterations. Other super-parameters were kept the same with SSD. All the training tasks were based on Titan V and Caffe framework [40].

## 3. Experiments and results

### 3.1. SSD-GPNet for gastric polyp images dataset

**Fig 5:**
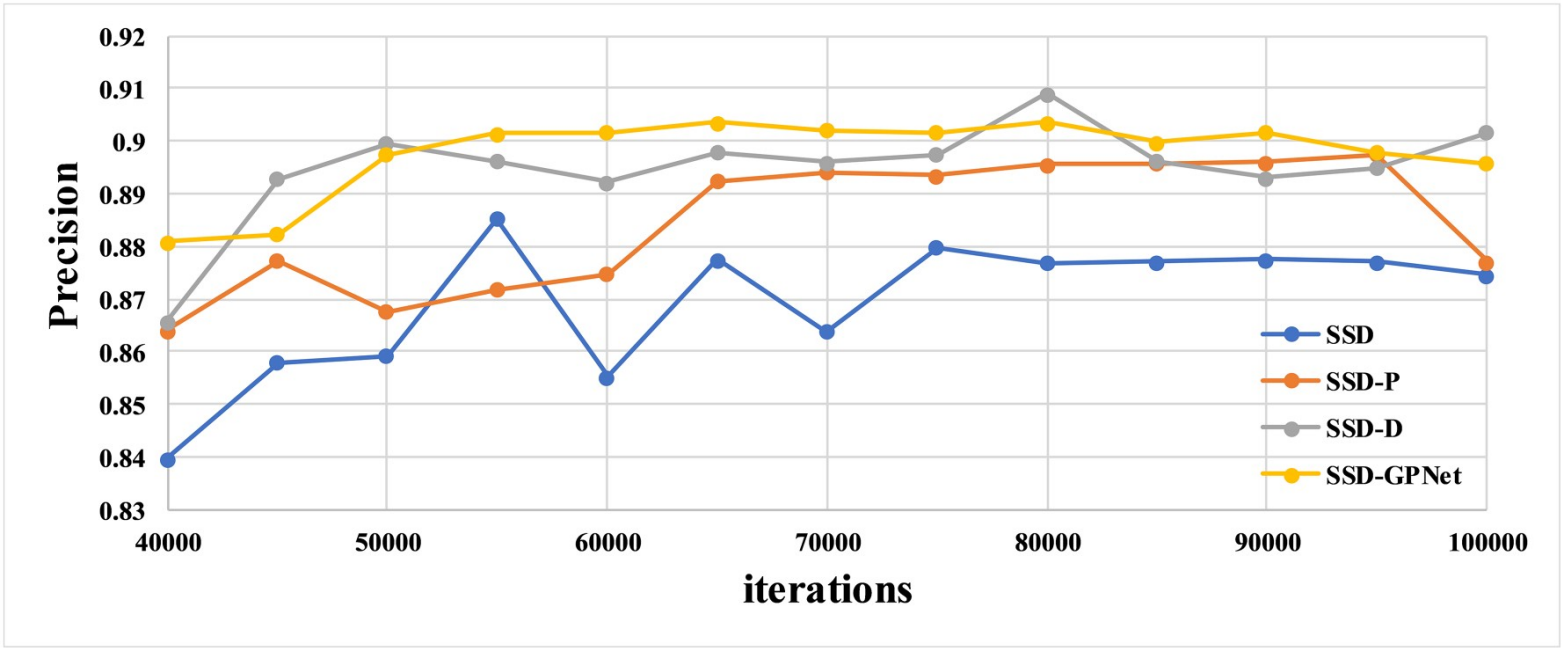
The results of gastric polyp detection on the test dataset using different network architectures trained only by gastric polyp images. The blue curve represents SSD. The orange curve represents SSD-P. The gray curve represents SSD-D. The yellow curve represents SSD-GPNet.

We conducted four experiments to detail the advantages of our SSD-GPNet. Each experiment corresponded to a network, namely, conventional SSD (SSD), SSD plus the pooling modules(SSD-P), SSD plus the deconvolution modules (SSD-D), and SSD plus the pooling and deconvolution modules (SSD-PD). SSD-PD was also called SSD-GPNet. Fig 5 shows the performance of trained models on the test dataset at different training iterations while training after 40,000 iterations. We concluded that the best mAP of SSD, SSD-P, SSD-D, and SSD-GPNet to detect gastric polyps were 88.5%, 89.7%, 90.9% and 90.4% respectively. SSD-GPNet did not achieve the highest mAP. The possible reason for this phenomenon was the lack of training images, and it was not easy to stabilize during model training. However, SSD-GPNet performed more stable than the other three network structures and SSD-GPNet had nearly 2% improvement compared to SSD.

**Fig 6:**
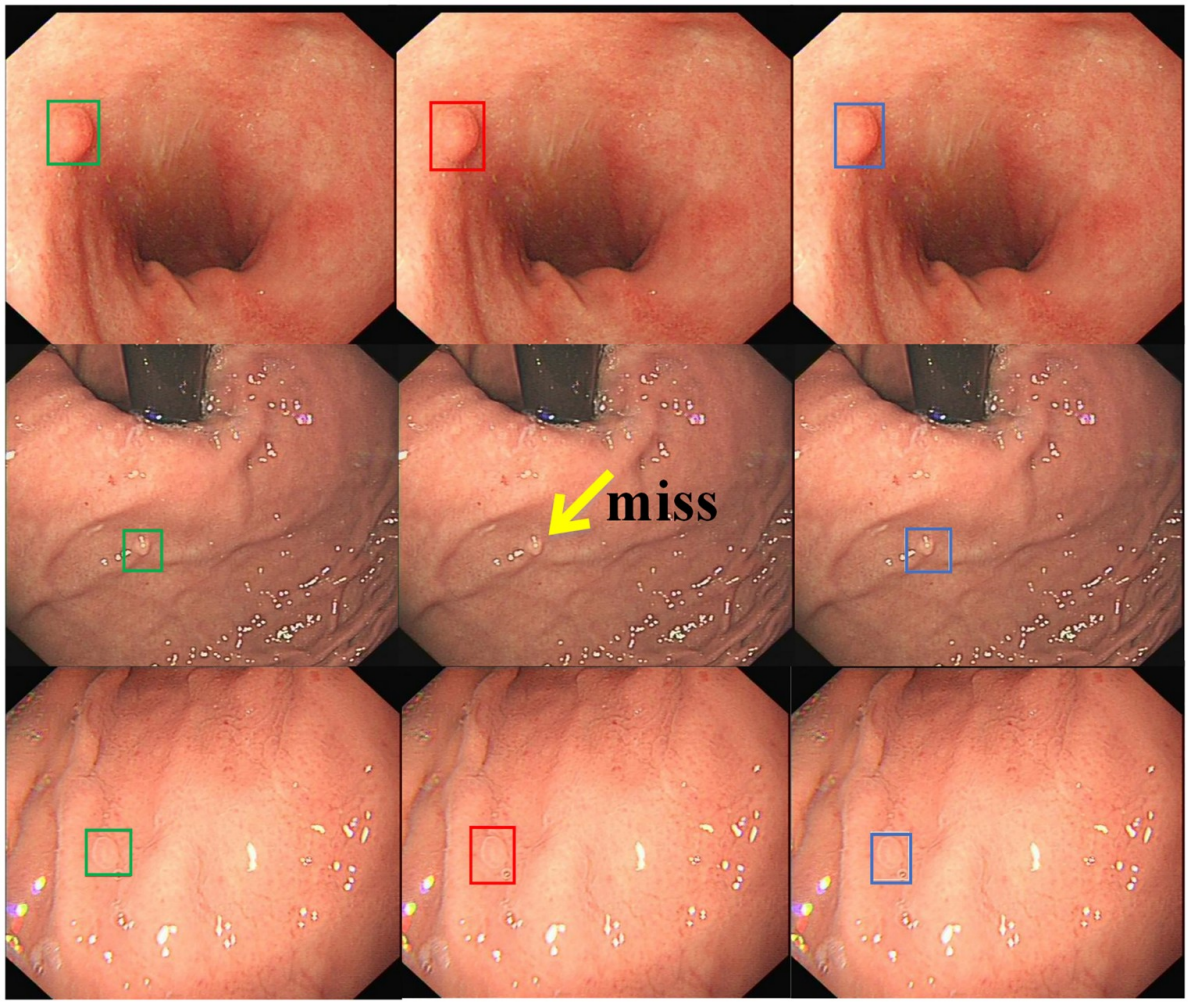
Some examples of detection results by conventional SSD and SSD-GPNet. The first column shows "ground truth" as labeled by experienced doctors. The second column shows SSD detection results. The last column shows SSD-GPNet detection results.

Fig 6 lists some examples of detection results by conventional SSD and SSD-GPNet. The first row is an example to depict that large polyps could be detected well by both networks. However, if the size of the polyp was small, SSD-GPNet performed better than conventional SSD (see the example of the second row; conventional SSD missed the detection of the small polyp), which meant that SSD-GPNet could extract and recognize more details of images. From the example of the third row, we can clearly see that the detection box of SSD-GPNet was more accurate than SSD.

### 3.2. SSD-GPNet for PASCAL VOC2007 and VOC2012

We have also conducted the training experiments only on the PASCAL VOC2007 and VOC2012 data [41] to further illustrate the fact that SSD-GPNet can improve the mAP of detection, not only in the area of medical images, but also in the field of nature images. The VOC dataset consists of 1 background class and 20 object classes with the annotated "ground truth" location and the corresponding class information for each image. Table 1 shows the comparison of SSD and SSD-GPNet results for the VOC dataset. The training dataset was part of VOC2007 and all of VOC2012; the test dataset was the rest of VOC2007. We found that SSD-GPNet improved the mAP by 2.1% compared with conventional SSD, and the mAP of almost all the classes had different degrees of improvement. All the statistics shown in Table 1 were tested by ourselves except the first row which came from [23].

**Table 1:** Results on VOC2007 test dataset using SSD and SSD-GPNet.

Method	Train	mAP	aero	bike	bird	boat	bottle	bus	car	cat	chair	Cow	table	dog	horse	mbike	person	plant	sheep	sofa	train	tv
SSD [23]	07+12	74.3	75.5	80.2	72.3	66.3	47.6	83.0	84.2	86.1	54.7	78.3	**73.9**	84.5	85.3	82.6	76.2	48.6	73.9	76.0	83.4	74.0
SSD	07+12	74.8	78.3	82.3	75.0	67.1	47.0	83.3	84.9	86.6	55.9	78.1	71.7	83.6	85.1	81.9	78.2	48.2	75.0	76.2	86.2	72.8
SSD-GPNet	07+12	**76.9**	**78.9**	**85.7**	**77.5**	**69.2**	**49.2**	**84.2**	**85.8**	**87.6**	**60.1**	**82.8**	73.0	**85.4**	**86.8**	**85.3**	**78.7**	**49.0**	**77.6**	**80.2**	**86.3**	**75.7**

### 3.3. Combining gastric polyp images with PASCAL VOC2007 and VOC2012

**Fig 7:**
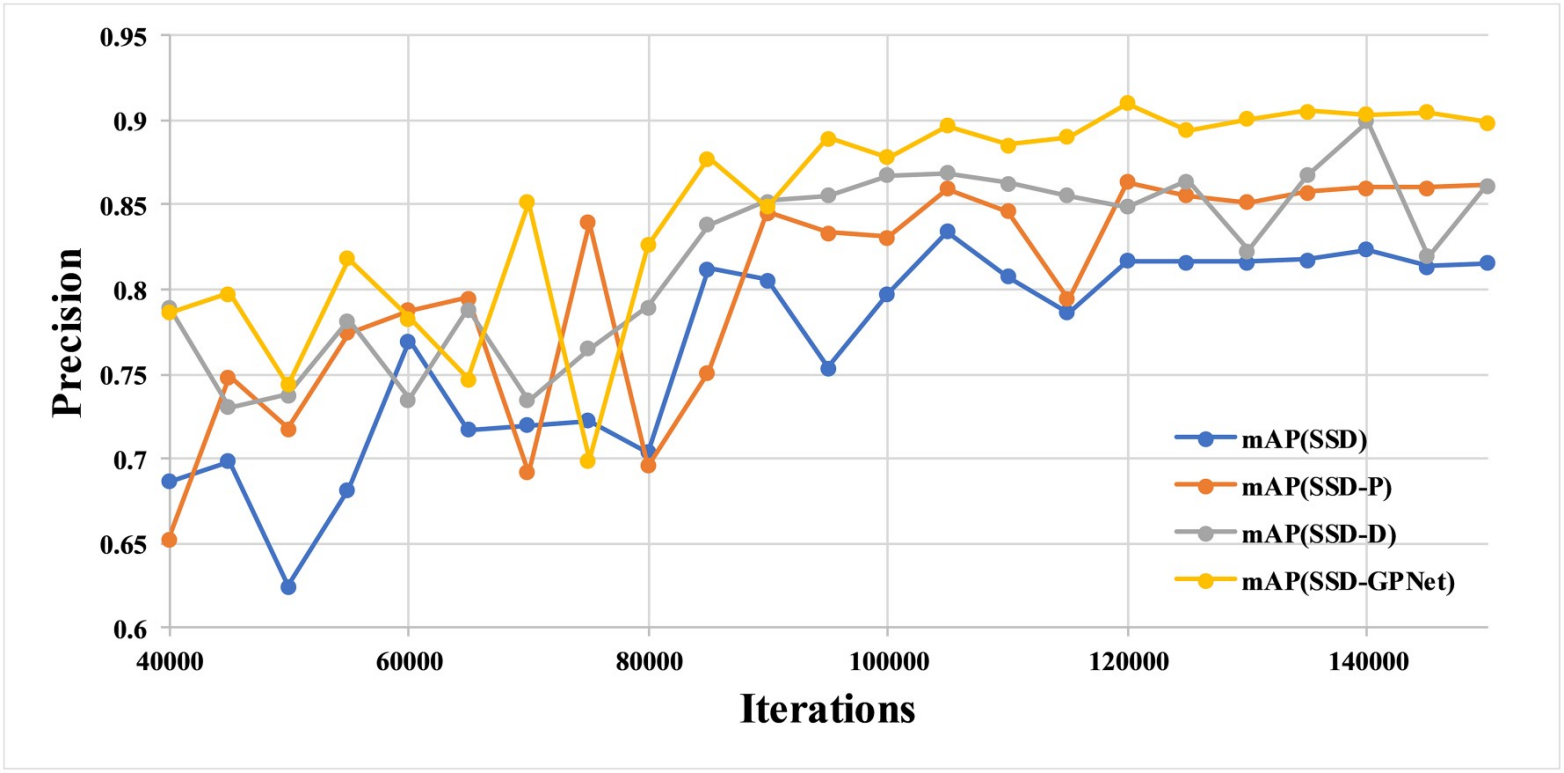
The mAP of gastric polyp detection of different networks trained by the combination of gastric polyp images and VOC dataset.

Our gastric polyp images numbered only 758 in total after data augmentation, which might be too few for deep learning. Generally, CNNs work well if there is a large amount of labeled data [42]. To verify that 758 images were enough for training and that the results in Section 3.1 are reliable, we conducted several further experiments. Considering that both the gastric images and the natural images were true color images, we created a new dataset including gastric polyp images and the VOC dataset. Because the VOC dataset consists of 1 background class and 20 object classes, we defined the “polyp” as the No. 21 object class for the VOC data. This alteration remedied the problem of small number of training images. To ensure that gastric images fully participated in the update of model parameters, we modified the iterations to 150,000. The base learning rate was 0.001, which would multiply 0.1 after 80,000 and 120,000 iterations. Fig 7 shows the performance of the models for detecting gastric polyps trained by gastric polyp images and VOC data using SSD, SSD-P, SSD-D, and SSD-GPNet networks. We determined that SSD-GPNet achieved the best mAP, 91.0%, which was even higher than the 90.4% depicted in Fig 5. Also, beyond 80,000 iterations, the curve of SSD-GPNet was less jagged than the other curves. The results reported in this section show that the results reported in Section 3.1 are reliable, implying that the SSD-GPNet we proposed is effective and more powerful than other methods.

### 3.4. Evaluation of gastric polyp detection performance

1. data description: We have also collected another 171 images from 72 patients at Sir Run Run Shaw Hospital in Zhejiang province in China. Each image had at least one polyp, and there were 182 polyps in total which were labeled by an experienced endoscopist. The processed image size was 560×475. Then we resized these images to 300×300 with Lanczos-4 interpolation algorithm based on 8×8 area to fit SSD-GPNet input size.
2. performance metrics: We introduced three basic metrics and four aggregation metrics to indicate different networks’ gastric polyp detection performance. Namely:
  - True Positive (TP): the networks correctly predicted polyp location. We set the threshold of Intersection over Union ratio (IoU) as 0.5. If IoU of ground truth bounding box and predicted bounding box was greater than 0.5, the polyp was correctly predicted.
  - False Positive (FP): the networks indicated a polyp location which did not correspond to a polyp.
  - False Negative (FN): the networks did not indicate a polyp location inside the polyp in the image, or if IoU of ground truth bounding box and predicted bounding box was smaller than 0.5, it was also a FN example.
  - Precision. Precision was calculated by (Eq 1).
  - Recall. Recall was calculated by (Eq 2).
  - F1-score. F1-score was calculated by (Eq 3).
    $$Precision=100*\frac{TP}{TP+FP}$$ (1)
    $$Recall=100*\frac{TP}{TP+FN}$$ (2)
    $$F1-score=\frac{2*Precison*Recall}{Precision+Recall}$$ (3)
3. detection results: Tables 2 and 3 listed the results of gastric polyp detection (the threshold was set as 0.5) with the dataset described in Section 3.4.

**Table 2:** Detection results with the threshold as 0.5.

Methods	SSD-GPNet	Conventional SSD
TP	**139**	121
FP	9	**7**
FN	**43**	61
Precision	93.92%	**94.53%**
Recall	**76.37%**	66.48%
F1-score	**84.24%**	78.06%

**Table 3:** The ability of two models to detect 182 polyps.

Conventional SSD	SSD-GPNet		Total polyps
	Correct	Wrong	
Correct	117	4	121
Wrong	22	39	61
Total polyps	139	43	182

**Fig 8:**
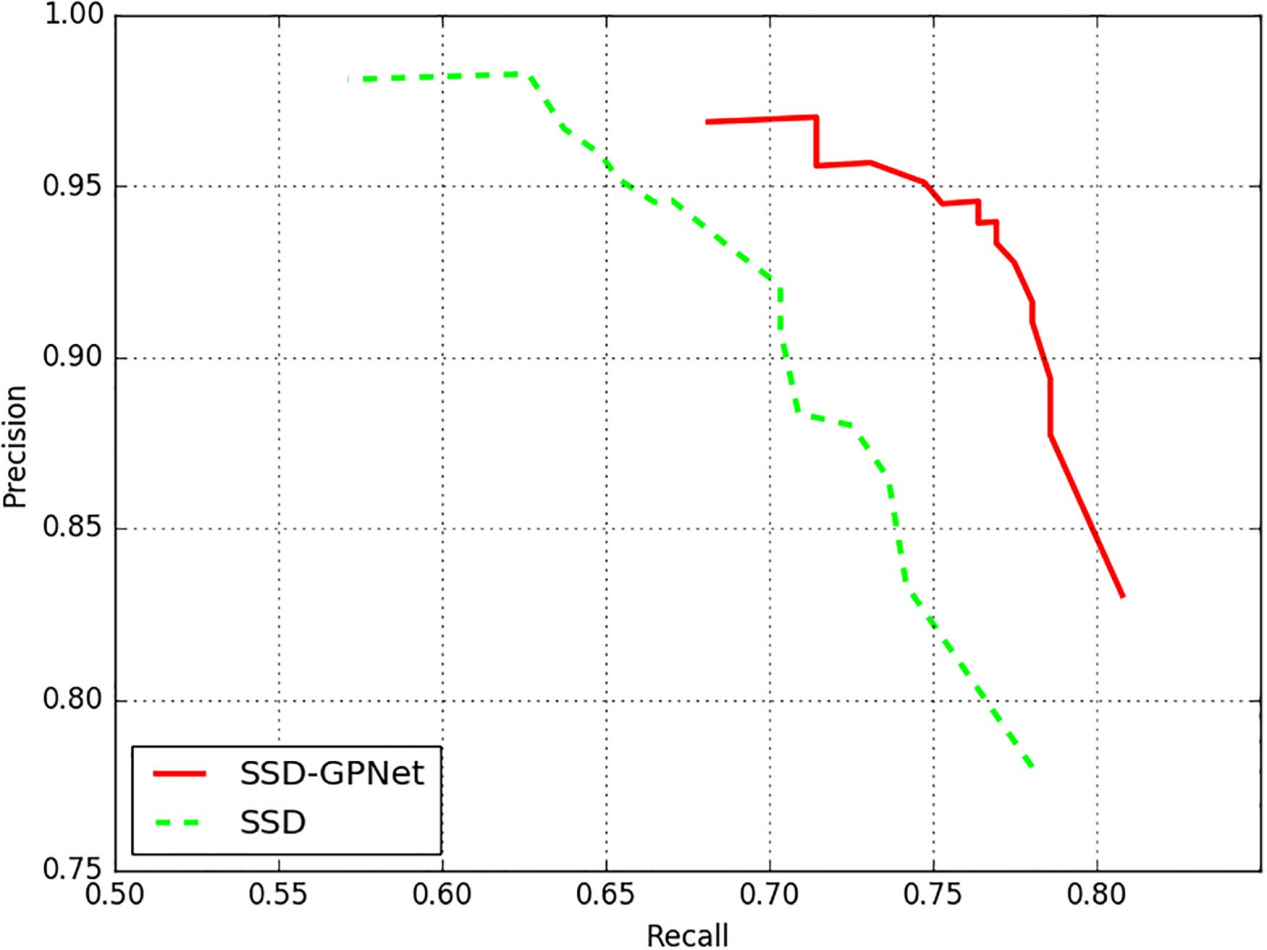
The comparison of P-R curves with different detection confidence thresholds between SSD-GPNet and SSD.

From Table 2, we could clearly find that the recall of SSD-GPNet was apparently higher than original SSD for nearly 10%. And SSD-GPNet could detect more true positive results without excessively increasing false positive results. In other words, SSD-GPNet performed better than conventional SSD to avoid polyp detection miss rate. The detection confidence threshold of SSD-GPNet and SSD was set as 0.5. Fig 8 showed the precision/recall (P-R) curve to better highlight the results with different thresholds on the polyp detection between the proposed method and SSD. The thresholds were set as 0.05, 0.10, 0.15, 0.20, 0.25, 0.30, 0.35, 0.40, 0.45, 0.50, 0.55, 0.60, 0.65, 0.70, 0.75, 0.80, 0.85, 0.90, 0.95, respectively. From Fig 8, we could find that our proposed network performed better than conventional network. To confirm the significance of the accuracy, a McNemar’s test [43] was conducted according to Table 3 to prove that SSD-GPNet performed better than conventional SSD (p = 0.00053).

Table 4 showed the details of recalls from different polyp size in pixels. The contents in parentheses denoted the recalls computed by the detected polyps and the total polyps in each corresponding domain. We divided polyp size in three levels (relative to the 560×475 image size instead of 300×300), namely small (area< = 32×32), medium (32×32<area< = 96×96), and large (area>96×96). We could find that more than half of the polyps’ area was smaller than 32×32. SSD-GPNet outperformed conventional SSD in detecting small and medium gastric polyps, which meant that SSD-GPNet had the ability to learn more detailed features to detect small and medium objects through the combination of multiple pooling methods and deconvolution operations. In clinical application, endoscopic physicians are more likely to miss small polyps. SSD-GPNet can help endoscopic physicians find small lesions to improve polyp detection rate.

**Table 4:** Recalls of different polyp size using different networks.

Methods	Small (area< = 32×32)	Medium(32×32<area< = 96×96)	Large (area>96×96)
SSD	0.5455 (54/99)	0.8026 (61/76)	**0.8571** (6/7)
SSD-GPNet	**0.6667**(66/99)	**0.9079** (69/76)	**0.8571** (6/7)

### 3.5. Comparison of time performance between SSD and SSD-GPNet

Because of the increasing feature maps in the feature pyramid’s layers, our SSD-GPNet was a little slower than conventional SSD. Our experiment was conducted on the combination of training and test datasets that contained 404 gastric polyp images. The GPU was Nvidia Titan V. Because we implemented the Sec_Max-Pooling, Sec_Min-Pooling, and Min-Pooling by ourselves, not using cuDNN v4 (which is not an open source software), our experiment reported in this section was based on Ubuntu16.04 + Caffe + GPU without cuDNN acceleration with Intel(R) Xeon(R) CPU E5-2609 v3 @ 1.90GHz. The results are shown in Table 5.

**Table 5:** Time performance comparison between SSD and SSD-GPNet without cuDNN.

Method	GPU	Model size (M)	Memory (M)	Time (s)	FPS (#)	Batch size	Class number	Input size
SSD (*)	Titan V	90.6	237.19	6.48	62	1	2	300×300
SSD-GPNet (*)	Titan V	134.9	347.77	8.06	50	1	2	300×300
SSD[23]	Titan X	-	-	-	46	1	21	300×300

(*) was tested by ourselves.

^(#)^ The values of FPS in this table do not include the time for real time interpolation from 570×475 to 300×300 image size.

From Table 5, we could easily see that due to the more complicated structure of SSD-GPNet, the model size increased nearly 50% with a sacrifice of 12 frames per second (FPS) compared to original SSD. However, SSD-GPNet was still able to achieve 50 FPS using Titan V, which meant that SSD-GPNet could achieve real-time gastric polyp detection to help endoscopists find polyps and relieve their workload.

## 4. Discussion and conclusion

In this paper, we have proposed an enhanced SSD called SSD-GPNet to detect gastric polyps, which can achieve real-time detection with 50 FPS using Titan V. To improve the mAP of detection, we proposed novel pooling methods which were applied to the feature pyramid network to reuse the lost useful information caused by Max-Pooling layers. At the same time, we have introduced a deconvolution operation to fully utilize the relationship between the layers in the feature pyramid network. Our SSD-GPNet improves the mAP about 2% while increasing the time required by only a small fraction. In addition, we applied SSD-GPNet to another collected dataset of gastric polyp images. The results show that SSD-GPNet greatly increase the polyp recall rate and can detect more true-positive polyps without excessively increasing false-positive results. SSD-GPNet is promising for gastric polyp detection in real time. It will help doctors find gastric polyps in future gastroscopy and avoid misdiagnosis.

The limitations of SSD-GPNet should be emphasized. First, the model trained by SSD-GPNet contains more parameters resulting in a slight decrease in time performance. Currently, we simply concatenate pooling results to increase the feature maps to avoid the loss of image information due to Max-Pooling. In the future, we will study how to fully utilize the image information without increasing the complexity of the networks. Second, the number of labeled gastric polyp images for training mentioned in this paper is small. In future scientific research, we will continue to increase the number of gastric polyp images for training to get more robust and stable polyp detection models. Third, gastric diseases also include ulcer, erosion, and early gastric cancer. In this paper, we only consider polyps as our detection target. In future work, we will be committed to implementing SSD-GPNet in detecting more gastric diseases.
